# Comparison of the White-Nose Syndrome Agent *Pseudogymnoascus destructans* to Cave-Dwelling Relatives Suggests Reduced Saprotrophic Enzyme Activity

**DOI:** 10.1371/journal.pone.0086437

**Published:** 2014-01-22

**Authors:** Hannah T. Reynolds, Hazel A. Barton

**Affiliations:** Department of Biology, University of Akron, Akron, Ohio, United States of America; University of California-Riverside, United States of America

## Abstract

White-nose Syndrome (WNS) is an emerging infectious mycosis that has impacted multiple species of North American bats since its initial discovery in 2006, yet the physiology of the causal agent, the psychrophilic fungus *Pseudogymnoascus destructans* ( = *Geomyces destructans*), is not well understood. We investigated the ability of *P. destructans* to secrete enzymes that could permit environmental growth or affect pathogenesis and compared enzyme activity across several *Pseudogymnoascus* species isolated from both hibernating bats and cave sediments. We found that *P. destructans* produced enzymes that could be beneficial in either a pathogenic or saprotrophic context, such as lipases, hemolysins, and urease, as well as chitinase and cellulases, which could aid in saprotrophic growth. The WNS pathogen showed significantly lower activity for urease and endoglucanase compared to con-generic species (*Pseudogymnoascus)*, which may indicate a shift in selective pressure to the detriment of *P. destructans’* saprotrophic ability. Based on the positive function of multiple saprotrophic enzymes, the causal agent of White-nose Syndrome shows potential for environmental growth on a variety of substrates found in caves, albeit at a reduced level compared to environmental strains. Our data suggest that if *P. destructans* emerged as an opportunistic infection from an environmental source, co-evolution with its host may have led to a reduced capacity for saprotrophic growth.

## Introduction

White-nose Syndrome is a fungal disease that has killed millions of hibernating bats in North America since its discovery in 2006 [1], [2]. The causal agent of WNS was identified as a novel fungal pathogen, *Geomyces destructans* [1], [3], [4], which was recently reclassified into the genus *Pseudogymnoascus* (Pseudeurotiaceae, *incertae sedis*, Leotiomycetes) [5]. While important details of physiology in this organism have been established such as growth temperature [6] and its ability to invade wing tissue [7], [8], many important aspects of its disease ecology remain unknown.

Invasive fungal pathogens are devastating plant and animal populations across the globe, and understanding their ecology is crucial in predicting disease outcomes [9]. The ability to carry out saprotrophic growth outside a host has important implications for disease management in emergent mycoses; models of host-pathogen dynamics indicate that high levels of saprotrophic growth can lead to host extinction [9]. For example, the emergent amphibian mycosis *Batrachochytryium dendrobatidis* (*Bd*) may be capable of growth outside its host, as it can be cultured on multiple carbon and nitrogen sources [10], including feathers [11]. Although definitive evidence of *Bd* environmental growth has not been obtained, models of the *Bd* infection cycle indicate that in addition to spore persistence, environmental growth could increase the risk of local host extinction [12]. The saprotrophic growth of *P. destructans* could permit year-round growth in caves, increasing both the chance of infection of naïve bats from an environmental reservoir, for which anecdotal evidence already exists (A. Hicks, pers. comm. 2011), and the risk of spread of this pathogen to new caves through contaminated sediment. Lorch et al. 2013 [13] found *Pd* DNA and viable *Pd* spores in cave sediments after the departure of the WNS-infected bat hosts; however, it is unknown whether the presence of *Pd* in these sediments is due to environmental propagation or to the persistence of spores shed from infected hosts. If the latter were the case, an obligate bat pathogen would be limited to growth in the hibernation season when bat body temperatures during torpor permit fungal growth, reducing the likelihood of environmental spread.

Current evidence suggests that *P. destructans* may be a facultative pathogen; it can grow on a variety of laboratory media, while obligate pathogens often cannot be cultured outside of their hosts on such media [4], [14]. While the current evidence is consistent with an introduction from Europe, the caves in which North American bats hibernate contain diverse, presumably native *Pseudogymnoascus* and *Geomyces spp.* [5], [16]–[19]. However, their role in the cave environment remains unknown [17], [19]. The partially annotated *P. destructans* genome indicates the presence of numerous genes coding for saprotrophic enzymes, including cellulases and chitinases [15]. If these enzymes confer a functional phenotype, they could allow *P. destructans* to grow saprotrophically in cave microhabitats and provide an environmental reservoir for infection of susceptible hosts. Questions regarding saprotrophic *Pseudogymnoascus* enzyme activity and how it compares to the activity of *P. destructans* may not only indicate the potential of an environmental reservoir for White-nose Syndrome, but may help us understand the natural history of this pathogen.

In this research, we examined enzymes that could play a role in fungal growth on various cave microhabitats, some of which could also be exploited in pathogenesis. We compared enzyme activity of *P. destructans* with that of a suite of *Pseudogymnoascus spp.* isolated from cave environments and the close relative *P. pannorum* var. *pannorum*. As additional controls, we included the established saprotroph *Penicillium pinophilum* (Trichocomaceae, Eurotiales, Eurotiomycetes), and *Oidiodendron maius* (Myxotrichaceae, *incertae sedis*, Leotiomycetes), which can form mutualistic associations with ericoid plants and has also been found as a saprobe in multiple locales [20], including cave sediments [16]. Our results indicate that *P. destructans* produces several enzymes that could permit environmental growth and support pathogenesis, but that *P. destructans* shows lower saprotrophic potential than closely related environmental isolates.

## Materials and Methods

### Cultures and Growth Conditions

Unless otherwise noted, all reagents were purchased from Sigma Aldrich (St. Louis, MO). *Pseudogymnoascus* species were isolated from Cudjo’s Cave, Cumberland Gap National Historic Park (NPS collection permit # CUGA-2009-SCI-0007) by swabbing hibernating little brown bats (*Myotis lucifugus*) and cave surfaces with pre-moistened (dH_2_O) sterile cotton swabs. This was followed by swabbing on Emmon’s modification of Sabouraud dextrose agar (SDA) augmented with cycloheximide (100 mg/L) [21]. Cultures were incubated at 5°C to select for psycrophilic and psychrotolerant *Pseudogymnoascus* species, and a library of 668 isolates was generated. Preliminary identification was performed using colony morphology and microscopy, and PCR amplification and sequencing of the internal transcribed sequence (*ITS*), which is considered the most robust genetic sequence for phylogenetic inference at the species level in the fungi [22], was used to confirm the identity of putative *Pseudogymnoascus spp.* and to assess the phylogenetic diversity of strains used in physiological assays. The ZR Soil Microbe DNA Miniprep Kit (Zymo Research, Irvine, CA) was used to extract DNA from cultures. Samples were prepared for PCR using 20–50 ng of template genomic DNA, 2X Taq Master Mix (New England Biolabs, Ipswich, MA), and 500 nM each of the primers ITS5 and ITS4 in a total volume of 20 µL [23]. PCR conditions were: initial denaturation at 94°C (2 min), 36 cycles of denaturation at 94°C (45 s), annealing at 52°C (45 s), elongation at 72°C (1 min 30 s), and a final extension at 72°C (10 min). To assess the diversity of strains used in enzyme analysis, two protein-coding genes were amplified: the transcription elongation factor EF1-α (*TEF1*) using the primers 983F/2218R [24] and DNA replication licensing factor MCM7 (*MCM7*) using the primers *Mcm7-*709for/Mcm7-1348 [25]. PCR conditions were the same as for the *ITS*, with 200 nM of each primer and the Taq MasterMix was used at 1.2X concentration. Collection information and Genbank accession numbers for the strains used in physiological tests are provided in Table 1, and full primer sequences are available in Table S2.

**Table 1:** Origin of isolates used in this study*.

Isolate	Sp.	Collection	Origin	Location	Test	ITS	MCM7	TEF1
**BL308**	1	Barton	*Mlf*	Tennessee, USA	10x	KF686750	KF686779	KF686764
**BL549**	1	Barton	*Mlf*	Tennessee, USA	10x	KF686751	KF686771	KF686767
**BL578**	1	Barton	*Mlf*	Tennessee, USA	10x	KF686752	KF686772	NA
**BL606**	1	Barton	*Mlf*	Tennessee, USA	10x	KF686753	KF686780	NA
**MYA 4855**	3	ATCC	*Mlf*	New York, USA	10x	KF686759	KF686773	KF686768
**16222**	2	ATCC	wfs	Germany	10x	NA	KF686777	KF686766
**MYA 4765**	4	ATCC	*Vmr*	Poland	10x	NA	NA	NA
**9644**	5	ATCC	radio	Papua New Guinea	10x	NA	NA	NA
**FI204**	1	Barton	cs	Tennessee, USA	3x	KF686754	NA	KF686763
**FI590**	1	Barton	cs	Tennessee, USA	3x	KF686755	NA	NA
**FI606**	1	Barton	cs	Tennessee, USA	3x	KF686756	NA	NA
**FI609**	1	Barton	cs	Tennessee, USA	3x	NA	KF686778	KF686761
**FI687**	1	Barton	cs	Tennessee, USA	3x	KF686757	KF686775	KF686769
**FI698**	1	Barton	cs	Tennessee, USA	3x	KF686758	KF686776	KF686765
**13PA1**	1	FCMR	cs	Pennsylvania, USA	3x	NA	KF686774	KF686770
**4NY16**	1	FCMR	cs	New York, USA	3x	JX270377	KF017653	KF017762
**4NY17A**	1	FCMR	cs	New York, USA	3x	JX270378	KF017654	KF017763 KF017763
**5NY8**	1	FCMR	cs	New York, USA	3x	JX270387	KF017656	KF017765 KF017765

* **Species codes:** 1 Pseudogymnoascus sp.; 2 Pseudogymnoascus pannorum,; 3 Pseudogymnoascus destructans; 4 Oidiodendron maius; 5 Penicillium pinophilum. **Origin codes:** cs, cave sediment; Mlf Myotis lucifugus fur; rs radio set; wfs wheat field soil; Vmr Vaccinium myrtilis roots.

Lab strains used in analysis were compared to species isolated from bat hibernacula from Lorch *et al* [13] and sequenced in Minnis *et al* [5] (Table S1). Sequences were visually inspected and assembled in Geneious version 6.0 [26] and aligned using MUSCLE version 3.7 [27] on the CIPRES server [28]. The alignment was visually examined in Mesquite version 2.75 [29] to exclude ambiguously aligned regions. Trees were generated for individual genes and a concatenated dataset, which included all taxa, even those with missing data. Evolution models were selected using the AIC criterion in jModelTest version 2.1.4 [30], [31], and were determined to be GTR+I+gamma for each of the three genes. Maximum likelihood trees were generated using RaxML [32] using the default settings plus GTRGAMMA+I for RAxML-HPC BlackBox version 7.27 on the CIPRES server; the best ML tree and 500 bootstrap replicates were produced in a single run.

For long-term storage at −80°C, spores were collected by washing sporulating cultures with 5 mL of 0.1% deoctylsulfosuccinate (DSS) in Sabouraud Dextrose Broth (containing 25% glycerol v/v) to suspend the hydrophobic spores. Five genetically distinct [5], unnamed *Pseudogymnoascus* species originally isolated by Lorch *et al*. [13] were obtained from the Center for Forest Mycology Research (CFMR; Madison, WI). Cultures of *P. destructans* ATCC MYA-4855 and *P. pannorum* var. *pannorum* ATCC 16222 were obtained from the American Type Culture Collection (ATCC, Manassas, VA), where they are stored under the prior name *Geomyces*. Control species, also obtained from the ATCC, were the ascomycetes *Penicillium pinophilum* ATCC MYA-9644 and *Oidiodendron maius* ATCC MYA-4765, an ericoid mycorrhizoid fungus in the Myxotrichaceae [33]. *Pseudogymnoascus*, though long thought to be a member of the Myxotrichaceae with *Oidiodendron* [34]–[36], has recently shown to be in a different Leotiomycete family [37]–[39], while *Penicillium* is a distantly related filamentous ascomycete in the class Eurotiomycetes [40]. Fungi were cultured at room temperature (20°C), except for *P. destructans*, which was cultured at 10°C on potato dextrose agar (PDA; 20 g/L dextrose, 4 g/L potato extract, 15 g/L agar, pH 6.8) from frozen spore suspensions prior to use in enzyme assays. For plate-based assays, point inoculation was used to transfer fungal spores from sporulating cultures to the center of prepared media. To inoculate filter paper cultures, spores suspensions (diluted to 10^7^ spores/mL) were prepared by washing cultures on PDA with 5 mL of DSS, which has previously been shown to be non-toxic to both *Geomyces* and *Pseudogymnoascus* species [41].

### Plate-based Assays

Enzyme tests in solid media were performed at pH 6.8 in agar-based media (15 g/L). To assess the temperature optima for *Pseudogymnoascus* growth, 10 replicates of each species were cultivated on PDA at 5, 10, 15, 20, 25, and 30°C, with the diameter of each colony checked weekly for six weeks. The weekly growth rate for each single replicate was calculated in using the lmList function in R version 2.15.1 [42], and the mean and standard deviation for each species at a given temperature was calculated. To assess enzyme activity, both the colony and clearance zone diameters were used to calculate the Relative Enzyme Activity (REA), which is the ratio of the clearance zone to colony size. To determine the best medium for hemolysis analysis, a pilot study was conducted on four media, each with 5% sheep’s blood: brain heart infusion agar and tryptic soy agar (TSA) from Hardy Diagnostics (Santa Maria, CA), and lab-prepared TSA and PDA. The PDA was prepared using 10 mL of PDA and 5 mL of a PDA/blood overlay. Based on the results of this pilot study, hemolysis was assessed at 10°C for the six *Pseudogymnoascus* species using TSA amended with 5% defibrillated sheep’s blood (Cleveland Scientific, Bath, OH). Lipase assays were performed on Spirit Blue Agar (BD Difco, Franklin Lakes, NJ); the agar mix was sterilized by autoclaving 20 min and cooled to 55°C before adding 30 mL of sterilized lipase reagent (100∶1 olive oil:Tween 80 in 400 mL deionized water). Colloidal chitin agar was prepared from shrimp shell chitin following the protocol in Hsu and Lockwood [43], with the following changes: the chitin was dissolved overnight rather than for 30 minutes in concentrated HCl, and was used immediately in agar preparation as a wet cake in sufficient quantity to yield 4 g/L dry weight. The production of urease was tested using Christensen’s urea agar [44]. To test cellulose degradation, media containing one of three different types of cellulose – carboxymethyl-cellulose (CMC), crystalline cellulose (Avicel), and D-cellobiose – were prepared following Yoon *et al* 2007 [45] with the following alterations: yeast extract was used rather than yeast nitrogen base, and dyes were used as post-stains based on Saczi *et al* [46] rather than being included in the media. Cellulase tests were repeated for *Pseudogymnoascus spp.* using yeast nitrogen base rather than yeast extract, with the cellulose as the sole carbon source. While the chitin, Avicel, Spirit Blue, and blood plates show distinct clearance zones, the CMC-agar and D-cellobiose agar require further processing. Based on *Saczi et al* [46], the CMC-agar was post-stained with Congo red for 1 hour and destained with 0.7 M NaCl for 15 minutes. Bromcresol purple (pH 6.8) was selected as a post-stain for D-cellobiose agar due to its ability to detect pH changes under mildly acidic to near-neutral conditions.

Humic and fulvic acids were extracted from potting soil as described in Bhullar *et al* [47] and used to prepare agar media with ∼20 mg/L of either humic or fulvic acid as the sole source of nutrients. Fungal cultures were inoculated using point inoculation and kept for one week at either 20°C or 10°C, with 10 replicates for each temperature/medium combination. Growth was recorded as the increase in colony diameter over time.

### Liquid Assays

Further tests of cellulase ability were performed in 50 mL liquid media with 9 cm Whatman #4 filter paper cut into 1 cm^2^ pieces. An inoculating dose of 10^6^ spores was added to this liquid assay, which also contained 0.1 M NaNO_3_, 11.48 µM K_2_HPO_4_, 7.35 µM KH_2_PO_4_, 6.10 µM MgSO_4_, 30 µM FeCl_3_, 1.24 µM ZnSO_4_, and 1.22 µM MnSO_4_. Two flasks were inoculated with *P. destructans* while one was used for each of the BL308, BL549, BL578, BL606, *P. pannorum* and *Penicillium pinophilum* cultures. After one month of shaking incubation (120 rpm) at 10°C, the cultures were centrifuged at 6000×*g*. The supernatant was filtered to remove cellular material and stored at 4°C prior to use in liquid cellulase assays. The presence of reducing and non-reducing sugars was assessed with a modification of the dinitrosalicylic acid (DNSA) reagent method from Miller [48]. Briefly, 2 mL of cellulose solution (1% CMC or Avicel in 0.05 M sodium citrate) and 2 mL of culture filtrate were incubated in a 50°C water bath for one hour. Samples were then subdivided; the first half of the sample was used directly to determine the presence of reducing sugars by adding 1 mL DNSA reagent, incubating at 95°C for 15 min, and adding of 1 mL of 40% potassium sodium tartrate. The other half of the sample was used to assess the presence of non-reducing sugars: before proceeding with the DNSA reagent, the sample was first hydrolyzed at 95°C for 5 minutes using HCl (final concentration: 0.24 M) and neutralized with KOH (final concentration: 0.25 M). Absorbance at 575 nm was measured using a DR2800 Spectrophotometer (Hach Co., Loveland, CO). Triplicate tests were performed for each culture filtrate and for control tests using either the enzyme solution or the cellulose solution in isolation, with standardized curves generated for glucose and sucrose using 0, 2.5, 5, 7, 9, and 10 mM solutions. Linear regression equations were used to calculate the mM of reducing and non-reducing sugars produced from each cellulase/enzyme assay.

The activity of β-glucosidase was assessed with the standard p-nitrophenyl-β-glucopyranoside (pNPβG) method. Briefly, 0.1 mL cellulose filtrate was added to 0.9 mL of a 0.02% solution of pNPβG in 0.1 M sodium acetate buffer (pH 4.8) and incubated at 50°C for 30 min before adding 2 mL of Clark and Lubs buffer (pH 9.8) [49] and measuring absorbance at 430 nm. A standard curve was prepared using 10^−1^, 10^−2^, 10^−3^, 10^−4^, 10^−5^, and 10^−6^ µM p-nitrophenol solutions, and the exponential regression equation was used to calculate the amount of p-nitrophenol released from pNPβG, an indication of β-glucosidase activity.

### Statistical Analysis

All statistical tests of enzyme activitiy were performed in R [42] using an ANOVA to test temperature and species effects and the Tukey’s Highly Signfiicant Differences (Tukey’s HSD) test to assess significant differences between each REA.

## Results

**Figure 1:**
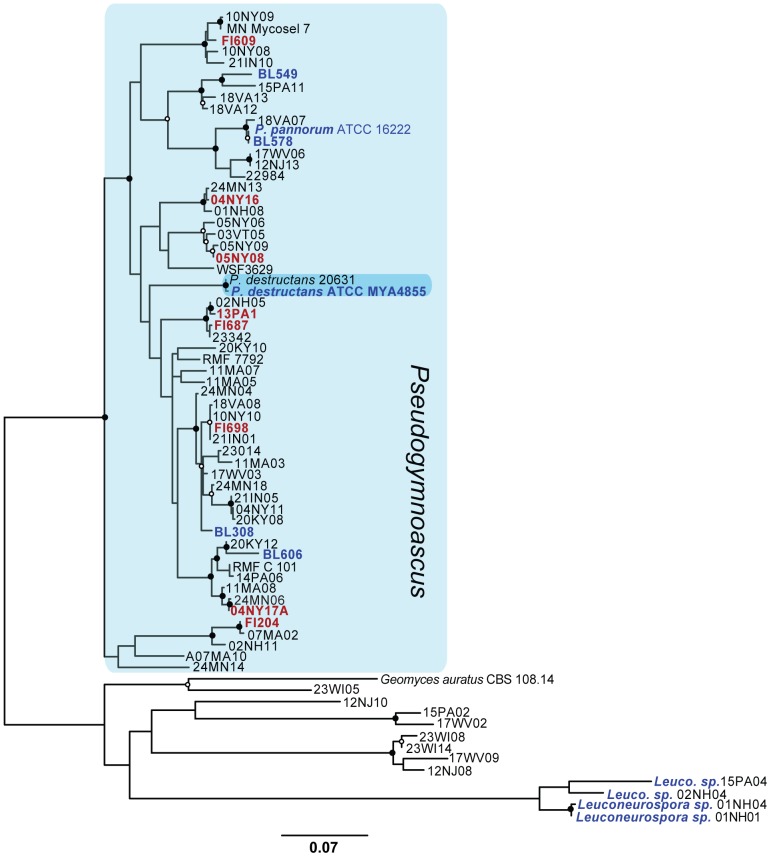
Best maximum-likelihood tree for the *Pseudogymnoascus spp.* used in this study (lnL = −10622.006982). A concatenated alignment of the TEF-1, MCM7, and partial ITS genes was used to generate a Maximum-likelihood tree in RAxML using strains identified in Minnis and Lindner [5]. Values show bootstrap support from 500 bootstrap replicates; closed circles indicate ≥95% support at each branch point, while open circles indicate >70% support. The taxa shown in red were used in 10X tests, while the taxa in blue were used in triplicate tests. *Geomyces auratus* and members of the *Leuconeurospora* were used as the outgroups. Accession numbers for each sequence can be found in Tables 1 and S1.

Before beginning our comparative enzymatic analyses, it was important to determine the relatedness of the bat and environmental *Pseudogymnoascus* isolates to each other and *P. destructans.* In order to do this, we used RAxML [32] to generate a phylogenetic tree of these isolates, including *P. destructans,* using a concatenated sequence of three genetic loci (ITS region: 876, *MCM7*: 486, *TEF1*: 806). This tree included 72 taxa (Tables 1 and S1), which allowed us to reliably place our identified isolates in each of the *Pseudogymonascus* clades [5]. The phylogeny (Figure 1) confirms the results of other investigators, in that *P. destructans* remains in a distinct clade from other North American *Pseudogymnoascus* isolates [4], [5]. The results also suggest that our environmental isolate BL578 is closely related to *P. pannorum* ATCC 16222, possibly as a subtype or strain.

Based on the phylogeny for our *Pseudogymnoascus* strains, two genetically diverse subsets of *Pseudogymnoascus spp* were selected for physiological tests: (1) a set of six *Pseudogymnoascus spp.* representing five clades, including four of our isolates (BL308, BL549, BL578 and BL606), *P. destructans,* the control species *P. pannorum*, *Penicillium pinophilum*, and *O. maius* (used in 10x replicate tests); and (2) a set of eight isolates from bat fur and cave sediments representing eight additional *Pseudogymnoascus* lineages (13PA1 to FI698) used in triplicate tests. The 10x set was used to permit statistical analysis comparing REA among species, while the 3x set was used to gain a broader assessment of the range of REA in *Pseudogymnoascus*.

**Figure 2:**
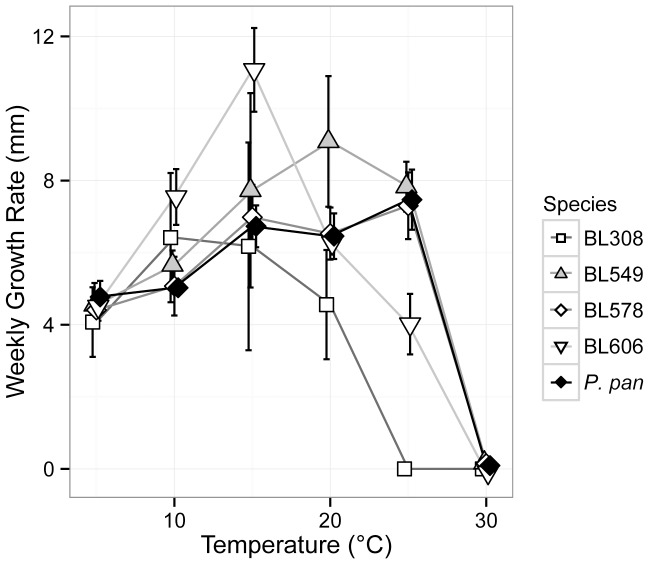
Temperature-dependent growth of environmental isolates of *Pseudogymnoascus*. The plot shows the average growth rate of each taxon over 6 weeks. Error bars indicate the significant difference of each assay (n = 10). *P. pan* = *P. pannorum.*

The optimal temperature of *P. destructans* growth ranges from 12.5–15.8°C, where it grows both radially and produces asexual spores, although growth on *Myotis lucifugus* bats in the environmental has been observed as low as 2°C [6]. To examine the role temperature might play in enzyme activity, we examined the growth of our strains at various temperatures and calculated the linear growth rates based on the weekly growth (Figure 2). All of the species examined grew between 5 and 25°C, with optimal growth varying between 10–25°C. Bat isolate BL308 was more restricted in its growth, showing an optimum growth of 10°C with no growth at 25°C, similar to *P. destructans*. Strains that were closely related to each other, such as BL578 and *P. pannorum* ATCC 16222, showed similar responses to temperature, but there was otherwise no immediately evident correlation between physiology and strain relationships. The bat-isolated strains in our study thus demonstrated both psychrophilic and psychrotolerant growth, in line with other members of *Pseudogymnoascus* and *Geomyces*. Based on these growth temperatures and our understanding of the temperature range of hibernacula (<12°C), we examined enzyme activity for the saprotrophic *Pseudogymnoascus spp.* at two temperatures: 10°C, which is close to the average temperature of bat hibernacula and the optimal growth temperature for *P. destructans* and strain BL308; and 20°C, the optimal growth temperature of some of our bat-isolated *Pseudogymnoascus* species. Using these temperatures allowed us to examine whether fungi show a higher REA at their optimal growth temperature or the temperature of the environment from which they were isolated.

**Figure 3:**
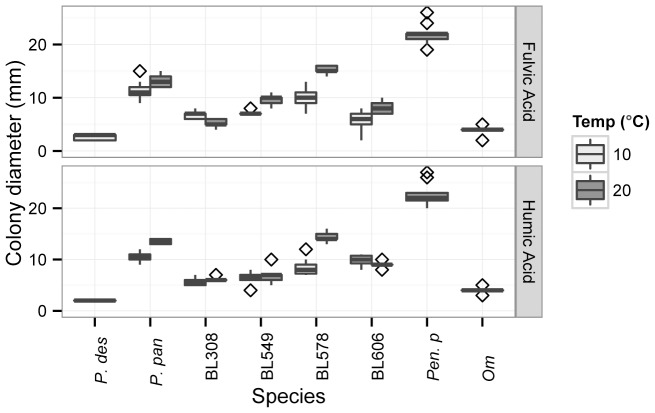
Growth of *Pseudogymnoascus spp*. on fulvic and humic acid extracts. Boxplot boundaries show the first and third quartiles, with the mean as the centerline and whiskers as 1.5 times the inter-quartile distance. Outliers are plotted as points. *P. des* = *P. destructans; P. pan* = *P. pannorum; Pen. p = Penicillium pinophilum;* and *Om = Oidiodendron maius.*

Fulvic and humic acids are complex aromatic and polyaromatic organic molecules rich in carboxyl and phenolate groups produced by cellulose decomposition in the environment [50]. We tested the ability of *Pseudogymnoascus spp.* to grow on these acids which, as the dominant form of dissolved organic carbon (DOC) in both soils and caves [51], [52], may support fungal growth in hibernacula. All fungi tested showed growth over one week on both fulvic and humic acid media; *P. destructans* and *O. maius* had the slowest growth rates, while the saprotroph *Penicillium pinophilum* had the highest growth rate (Figure 3). Both the temperature and species tested significantly affected growth rate on both fulvic and humic acids (ANOVA results in Table S3). A Tukey’s HSD test of the temperature and species effects found that *P. destructans* had significantly lower growth than most other *Pseudogymnoascus* species, but was not significantly different from environmental isolates BL308 (10°C and 20°C), BL549 (10°C), or from *O. maius* (10°C) (significance values are shown in Table S4). A comparison of psychrotolerant *Pseudogymnoascus* species at ambient (20°C) and cool temperatures (10°C) indicated that for most species, temperature did not affect growth rate on this medium, while strain BL578 and BL549 grew significantly better at 20°C than at 10°C on fulvic acid, and BL578 and *P. pannorum* grew significantly better at 20°C than at 10°C on humic acid (significance values are shown in Table S6).

**Figure 4:**
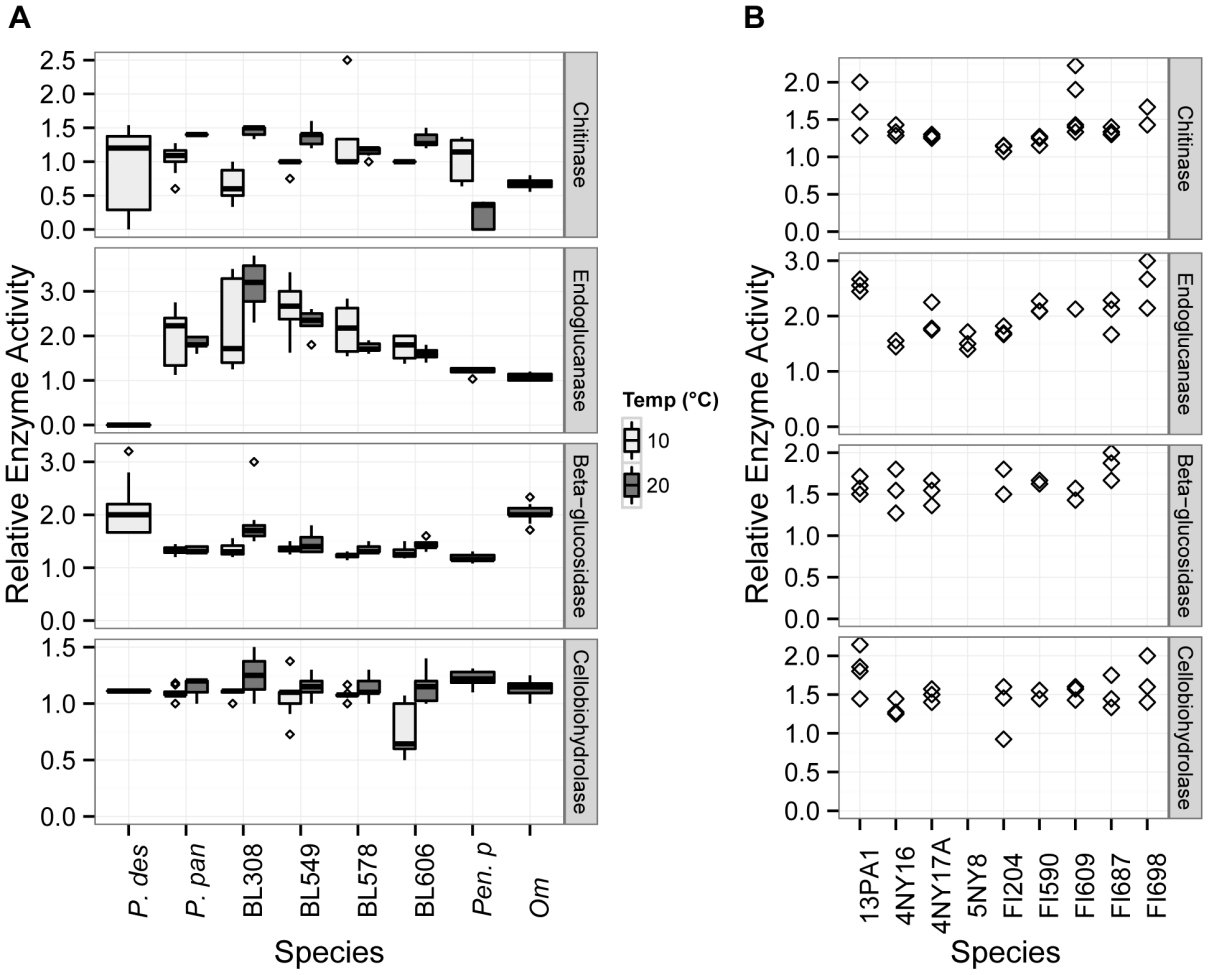
Relative enzyme activity for saprotrophic enzymes in *P. destructans* and other fungi. Boxplot boundaries show the first and third quartiles, with the mean as the centerline and whiskers as 1.5 times the inter-quartile distance. Outliers are plotted as points. *P. des* = *P. destructans; P. pan* = *P. pannorum; Pen. p = Penicillium pinophilum;* and *Om = Oidiodendron maius.*

The presence of cellulose and chitin in caves may be dependent on environmental effects that vary among sites, such as flooding or the presence of bat and/or insect populations [53], [54]; caves that experience periodical flooding can accumulate large quantities of plant debris [55] and the guano of insectivorous bats contains a high amount of chitinous insect cuticle [56]. We therefore investigated the ability of *P. destructans* to produce cellulases and chitinases (Figure 4), which could provide an environmental carbon and energy source for the growth of *P. destructans*. While most of the species examined could be assessed for chitinase at one week, the REA of *O. maius* and *P. destructans* were assessed at two and four weeks, respectively. All examined species produced chitinases, with *P. destructans* showing similar activity to a suite of related fungal species. Four of the five environmental *Pseudogymnoascus* isolates showed lower chitinase REA at 10°C than at 20°C, but this difference was significant only for BL308 (Tukey’s adjusted p-value <0.001; see Table S6 for other values). The lowest chitinase activity observed was for the *Penicillium pinophilum* at 20°C. At 10°C, *P. destructans* chitinase activity did not differ significantly from that of the other species (Table S5), but chitinase activity for *P. destructans* at 10°C was significantly lower than that of BL308 and BL549 and higher than that of *P. pinophilum* at 20°C. While *P. destructans* and *P. pinophilum* showed similar REA to the other fungi, the clearance zone for these two species remained slightly cloudy, suggesting an incomplete hydrolysis. Growth remained scant for these species, with little aerial mycelia.

Due to the potential role of plant detritus in promoting fungal growth in caves, we assessed the activity of three different functional categories of cellulases: endoglucanases, which cleave internal cellulose bonds, exoglucanases, which cleave the non-reducing end of the cellulose polymer, and β-glucosidases, which break down the disaccharide cellulose by-product. Due to varying growth rates, *P. destructans* was assessed for endoglucanase at three weeks, β-glucosidase at two weeks, and cellobiohydrolase at four weeks. The other *Pseudogymnoascus spp.* were assessed at one week for endoglucanase at both temperatures, while the REA of β-glucosidase and cellobiohydrolase was measured at one week for the 20°C assay and two weeks for the 10°C assay. *P. pinophilum* and *O. maius* were assessed at one week and two weeks, respectively, for all cellulase categories. The endoglucanase assay (using CMC) was negative for *P. destructans*, but positive for all other species examined (Figure 4). This assay did not yield the classic red-to-black color change that indicates medium acidification, but showed a pale red zone indicating where cellulose had been degraded surrounding colonies. When the tests were repeated using CMC as the sole carbon source, the REA increased for each species, but remained negative for *P. destructans.* Despite its apparent lack of endoglucanase activity, *P. destructans* was able to grow and sporulate on CMC media. The β-glucosidase assay indicated that *P. destructans* had significantly higher REA than all other fungi examined except for BL308 and the plant mutualist *O. maius*, which were not significantly different (Table S5). Cellobiohydrolase REA was positive for each fungus, and, though much slower to develop in *P. destructans*, not significantly different from the other fungi (Table S5), except BL606, which had significantly lower REA at 10°C (Tukey’s adjusted p-value = 0.001). Temperature was a significant factor overall for β-glucosidase (ANOVA F-value = 16.005, p-value <0.001) and cellobiohydrolase activity (ANOVA F-value = 35.871, p<0.001), but not for endoglucanase (ANOVA F-value = 0.004, p = 0.948). Nevertheless, BL308 showed significantly higher endoglucanase activity at 20°C than at 10°C (Tukey’s adjusted p-value = 0.001), as it did for β-glucosidase activity (Tukey’s adjusted p-value <0.001). BL606 was the only strain showing significant differences for cellobiohydrolase activity at these two temperatures, with higher activity at 20°C (Tukey’s adjusted p-value = 0.004) (Table S6).

**Figure 5:**
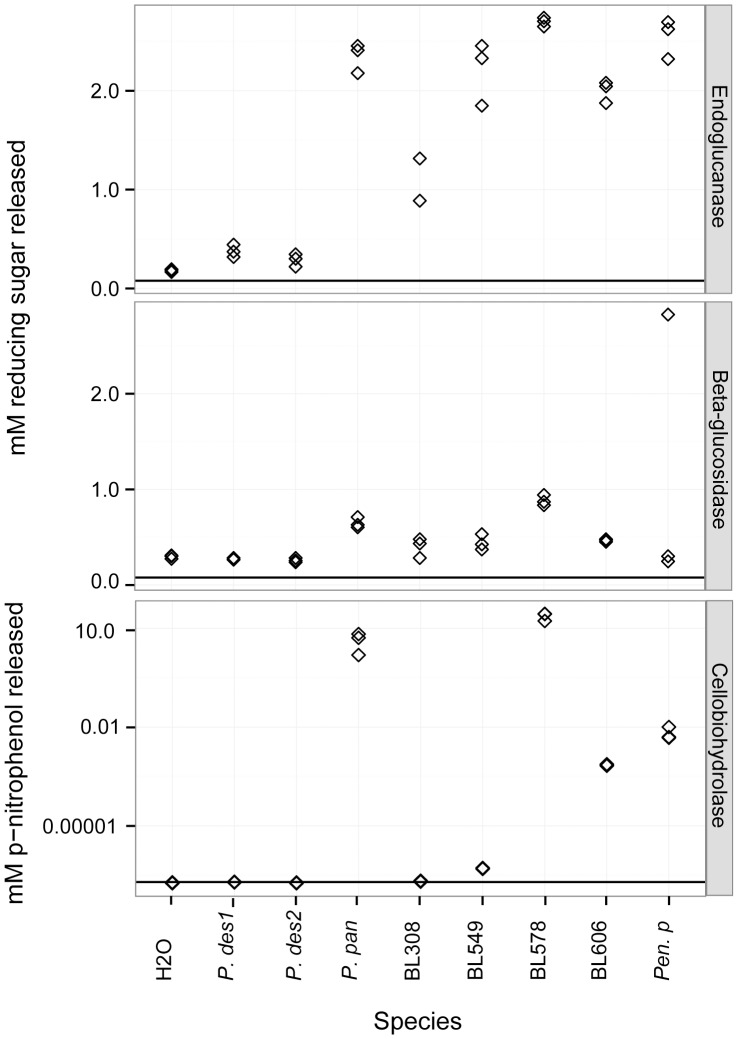
Liquid assays of endoglucanase, β-glucosidase, and cellobiohydrolase. Reducing sugars released from CMC (endoglucanase activity) and Avicel (β-glucosidase activity) when treated with fungal filter paper culture supernatant and p-nitrophenol release from pNPβG (cellobiohydrolase activity). Tests were performed in triplicate, and the results were plotted individually. The horizontal line indicates results for negative controls using culture filtrates and H_2_O.

To determine which cleavage products were released from cellulose for potential fungal growth, we examined the production of reducing and non-reducing sugars in liquid assays. Using CMC, these assays were negative for the production of non-reducing sugars by all the fungi tested (data not shown), while reducing sugars were produced (Figure 5). The *P. destructans* culture filtrate released a small amount of reducing sugars from CMC (mean ± SD = 0.333±0.008 µM), which was double that of the negative control (0.182±0.014 µM). The other fungi assayed released much higher levels of reducing sugars than *P. destructans*, with BL308 as the least active (mean = 1.03±0.247 µM) and BL578 as the most active (mean = 2.70±0.045 µM). A subsequent assay using the insoluble cellulose Avicel tested negative for reducing sugars using *P. destructans* filtrate (0.265±0.017 µM) compared to the negative control (0.295±0.019 µM). The other *Pseudogymnoascus spp.* tested positive for reducing sugars on Avicel, ranging from 0.398±0.103 µM (BL308) to 0.882±0.054 µM (BL578), while *Penicillium pinophilum* tested negative (0.274±0.038 µM). The cellobiohydrolase assay using pNPβG was negative for *P. destructans* and BL308, but positive for the other *Pseudogymnoascus spp.* and *Penicillium pinophilum* (Figure 5).

**Figure 6:**
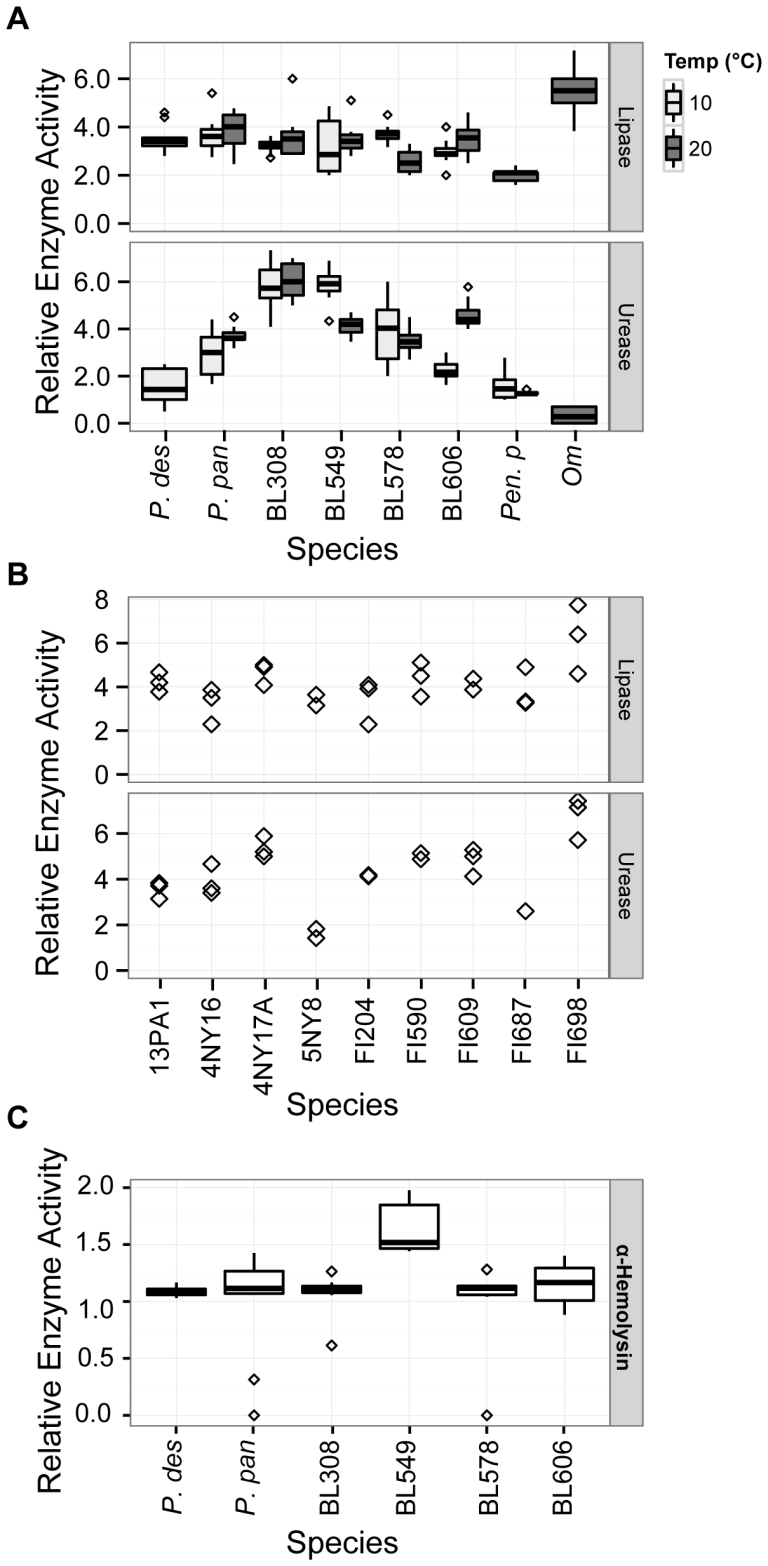
Relative enzyme activity of enzymes that may have pathogenic and saprotrophic function in environmental *Pseudogymnoascus spp.* and *P. destructans*. A) 10x replicates testing lipase and urease activity at hibernaculum and ambient temperature. B) 3x replicates testing lipase and urease activity at ambient temperature. C) 10x replicates testing α-hemolysis activity on on tryptic soy agar/5% sheep’s blood after 8 weeks (environmental isolates) and 11 weeks (*P. destructans*). Boxplot boundaries show the first and third quartiles, with the mean as the centerline and whiskers as 1.5 times the inter-quartile distance. Outliers are plotted as points.

In addition to examining potential saptrotrophic enzyme activity in our *Pseudogymnoascus* isolates, we compared them for enzymatic activity that could aid in pathogenesis. To determine whether *P. destructans* produces enzymes that could play a role in breaking down host tissues, we tested whether members of *Pseudogymnoascus* could produce lipases (Figure 6A & B) and hemolases (Figure 6C). We initially tested hemolysis on four complex media containing 0.5% sheep’s blood, each of which contain different levels of nutrients. Hemolysis was noted only in TSA/blood plates, and not in the BHI/blood or PDA/blood preparations (data not shown). Lipase REA was not significantly affected by temperature (ANOVA F-value = 0.002, p = 0.961), although REA for BL578 was significantly higher at 10°C than at 20°C (Tukey’s adjusted p-value = 0.025). While lipase activity was rapid (visible in one week for all species save *P. destructans* and *O. maius*, which were assessed at two weeks), hemolysis was slow. Alpha-hemolysis, which indicates the partial lysis of red blood cells, was observed in our fungal cultures only after several weeks: 11 weeks for *P. destructans* and 8 weeks for the other *Pseudogymnoascus spp.,* which grew more rapidly. The REA of *P. destructans* α-hemolysis was not significantly different from that of the other *Pseudogymnoascus spp.* (Table S4). The complete lysis of red blood cells and breakdown of hemoglobin (β-hemolysis) was not observed for any of the tested fungi.

If degraded by urease, the urea in bat urine and guano could serve as a source of nitrogen for fungal growth. Urease could also play an important role in pathogenesis, as the enzyme has been implicated in virulence pathways in other pathogenic microorganisms [57]. We tested urease activity on Christensen’s urea agar, and found that all the fungi tested except *O. maius* rapidly produced the enzyme (Figure 6A & B). The 20°C assays were assessed at one week, but the 10°C assays for the *Pseudogymnoascus spp.* and *P. destructans* were measured at two weeks, save for BL606, which was assessed at one week. The plant mutualist *O. maius* developed weakly positive REA for urease only after seven weeks. The *Pseudogymnoascus spp.* showed wide variation in their urease ability, ranging from average REAs of 1.54 for *P. destructans* to 6.03 for BL308 (20°C). The *Pseudogymnoascus spp.* tended to show high urease activity, with some species producing urease zones over six-fold greater than the colony diameter. While *P. destructans* showed significantly lower urease activity than did all other examined *Pseudogymnoascus spp.*, it produced urease with an activity zone double its colony size, similar to BL606 at 10°C and *P. pannorum* at 10°C and 20°C. Temperature was not a significant factor in urease REA overall (ANOVA F-value = 2.839, p = 0.0947), but BL549 produced significantly higher urease REA at 10°C than at 20°C (Tukey’s adjusted p-value <0.001), while BL606 produced significantly higher urease REA at 20°C (Tukey’s adjusted p-value <0.001).

## Discussion

As top predators in their ecosystems, bats play a critical role in controlling insect populations. Thus, the high mortality in multiple bat species from White-nose Syndrome renders *P. destructans* a major threat not only to these wildlife populations, but also to agriculture and public health [1], [58], [59]. The recent findings of the persistence of *P. destructans* in cave sediments suggest that the fungus can survive in bat hibernacula even in the absence of its host, which has a profound impact on both disease management and the epidemiology of the disease [13]. We therefore compared the function of several enzymes that could serve in a saprotrophic niche for the pathogenic *P. destructans* as well as resident *Pseudogymnoascus* species. The enzymes selected for this study were not intended to be an exhaustive survey of *Pseudogymnoascus* metabolism; rather, they were chosen to represent enzymes that might be required for growth on major components of the cave ecosystem. We also examined a subset of enzymes that could have dual function for these fungi, supporting saprotrophic growth and potentially contributing to a pathogenic lifestyle.

Cave environments are generally carbon and energy-limited due to geologic isolation and the lack of light driven photosynthesis [60]. As a result, the substrates available to fungi in caves are primarily in the form of allochthonous input, such as soil-derived dissolved organic carbon (DOC) or detritus, including leaf litter from entering surface streams. Another source of energy is the organic waste of trogloxenes, such as crickets and other arthropods and bats and their chitin- and urea-rich bat guano [53]–[54]. We therefore assessed the activity of chitinase and cellulase activity in *Pseudogymnoascus*, as well as the ability to grow on DOC in the form of fulvic and humic acids. Our results suggest that while *P. destructans* demonstrates enzymatic activity in each of these groups, testing positive for growth on fulvic and humic acids (Figure 3) and for chitinase and cellulase activity (Figure 4), it shows slower growth and lower cellulase activity relative to other *Pseudogymnoascus spp.* The chitinase relative enzyme activity in *P. destructans* was similar to other tested *Pseudogymnoasci,* while actual growth of the pathogen on chitin was limited. The chitinase production by other *Pseudogymnoasci* indicates that these species can use chitin as a nutrition source, which may allow growth on insects and/or the insect remnants found in bat guano, although such growth by *P. destructans* would likely be comparatively slow.

Assays of cellulases that would allow growth on plant debris were contradictory. In plate assays, *P. destructans* showed the highest β-glucosidase REA of all examined species, but was slow to develop cellobiohydrolase and was negative for endoglucanase (Figure 4). Liquid assays found that *P. destructans* using filter paper as a source of carbon produced endoglucanases, but not β-glucosidases or cellobiohydrolases (Figure 5). The negative result on solid media for endoglucanase may have been due to the assay’s reliance on visible color differences in the surrounding media, which may thus have a higher minimum detection limit than the liquid assay. Strain BL308 and *Penicillium pinophilum*, like *P. destructans*, tested negative in liquid tests for Avicel, a synthetic microcrystalline cellulose, but positive for growth on the substrate on solid media. These results suggest that *P. destructans* does produce each class of cellulase, but only to a limited degree and under restricted conditions. Compared to the bat-isolated and environmental *Pseudogymnoascus spp.*, *P. destructans* appears capable of degrading cellulose, albeit at a reduced capacity.

Although occasional, superficial mycoses in humans and animals by members of *Pseudogymnoascus* have been observed, members of this genus are considered to be predominately saprotrophic [61], [62]. To assess the expression of enzymes that might function in either a saprotrophic or pathogenic context, we examined lipase, hemolase and urease activity. Our data demonstrated that *P. destructans* was positive for all three enzymes, with lipase activity similar to the other members of the genus (Figure 6). Lipolytic fungi are known to play an important role in the beginning stages of leaf litter decomposition [63], and lipases can also aid infectious mycoses in host tissue adhesion and invasion [64]. All *Pseudogymnoascus spp.* examined were positive for α-hemolysis after several weeks of incubation, although *P. destructans* hemolysis occurred more slowly. Compared to the rapid hemolysis seen in true pathogens, such as the bacterial pathogens *Staphylococcus aureus* and *Streptococcus pyogenes,* which generate visible zones of hemolysis on blood agar within hours [65], [66], the hemolytic activity in *P. destructans* appears to begin when nutrients become limiting. Thus, hemolytic activity by *P. destructans* may not be optimized for systemic growth within its host, but rather may aid in survival on the nutrient-limited landscape of a bat wing membrane. This high lipase activity but low hemolysin activity corresponds with the histological evidence of WNS infection, where the fungus degrades the wing epithelium and basal membrane to invade connective tissue [8], [67], but does so without directly invading the blood vessels [67]. Thus, hemolysis may not play an important role in disease pathology, but may be necessary to obtain the iron needed to support fungal growth.

All of the fungi examined in this study save *O. maius* showed high urease activity. Urease would be advantageous for growth in bat guano, but as this enzyme is broadly distributed among soil microbes [68], [69], urease proficiency is probably not a specific adaption to cave life. Nonetheless, urease has been implicated in tissue invasion and virulence signaling pathways [57], [70], [71], including in *Coccidioides posadasii,* where ammonia produced by urease damages host tissues [72], [73] and *Cryptococcus neoformans,* where urease plays an important role in crossing the blood-brain barrier [74]. Urease may thus play two roles in WNS: permitting propagation of *P. destructans* on guano and potentially aiding in pathology. Further research is needed to understand the possible involvement of these enzymes in WNS disease ecology, as well as other saprotrophic pathways that may have been coopted for pathogenesis.

We had examined the effect of temperature on enzyme activity to test whether species would show higher REA at their optimal growth temperature or at the temperature of the environment from which they had been isolated. In fact, we found that temperature rarely caused a significant difference in relative enzyme activity, and in the majority of cases that did show a significant difference, the REA was higher at 20°C, regardless of the temperature preferences of the organism or site of origin. For instance, BL308, which has an optimal growth at 10°C and was isolated from a cold cave, showed significantly higher chitinase, endoglucanase, and β-glucosidase activity at 20°C. The only two instances of significantly higher REA at 10°C were for BL549 urease and BL578 lipase; these two species have optimal growth at 20°C and 25°C respectively. Thus, while some of the cave-isolated fungi grow more quickly at the cool temperatures found in northern bat hibernacula, their enzyme function indicates that they are not likely restricted to these environments.

The results of this study indicate that the *Pseudogymnoascus spp.* found in caves can degrade a variety of organic molecules for growth in multiple microhabitats, including insect detritus, decaying plant material, and bat guano. Such results suggest that rather than having a specific niche, members of the genus *Pseudogymnoascus* can function as generalist decomposers, a finding supported by the presence of *Pseudogymnoascus* and related *Geomyces* species worldwide [75]–[82], including Antarctica and the Arctic. Our finding that *P. destructans* shows similar saprotrophic potential to other members of this genus may indicate a potential environmental origin for this pathogen; however, the reduced growth rate and saprotrophic enzyme activity suggest that *P. destructans* may have gained functionality in pathogenesis at the expense of an environmental lifestyle. Such a diminished saprotrophic capacity could be explained if *P. destructans* had entered into an evolutionary host-pathogen relationship at sometime in the past; a significant length of coevolution between host and pathogen could have resulted in a decrease in the selective pressure on *P. destructans* to maintain enzymes needed for decomposition. To determine if this is indeed the case, a phylogenetic comparison of the genes involved in both saprotrophic and pathogenic pathways may be able to detect changes in either expression pathways and/or enzymatic activity and could potentially provide a molecular clock for the timing of the movement of *P. destructans* out of the soil/cave sediment environment and into its host.

## Supporting Information

Table S1 Collection information and Genbank accession numbers for strains used in assessing phylogenetic diversity.
(XLSX)

Table S2 Primers used for sequencing *Pseudogymnoascus* isolates.
(XLSX)

Table S3 Results of two-way ANOVA assessing temperature and species effects in relative enzyme activity.
(DOCX)

Table S4 Tukey’s Highly Significant Differences Test for growth on organic acids comparing *P. destructans* to other species.
(DOCX)

Table S5 Tukey’s Highly Significant Differences Test for relative enzyme activity comparing multiple fungal species to *P. destructans.*
(DOCX)

Table S6 Tukeys’ Highly Significant Differences Test comparing intraspecific growth or relative enzyme activity at 20°C and 10°C.
(DOCX)
