# Supplementary material for: Comparison of the White-Nose Syndrome Agent Pseudogymnoascus destructans to Cave-Dwelling Relatives Suggests Reduced Saprotrophic Enzyme Activity
Source: PLoS One. 2014 Jan 22;9(1):e86437. doi: 10.1371/journal.pone.0086437 (PMC3899275; doi:10.1371/journal.pone.0086437)
Supplement: Table S3 — Results of two-way ANOVA assessing temperature and species effects in relative enzyme activity. (DOCX) [file pone.0086437.s003.docx]

**Table S3.** Results of two-way ANOVA assessing temperature and species effects in relative enzyme activity.

| Enzyme | Factor | df | Sum of squares | Mean squares | F-value | p-value |
| --- | --- | --- | --- | --- | --- | --- |
| Fulvic Acid Growth | sp | 7 | 3115.1 | 445.0 | 262.2 | <0.001 |
|  | temp | 1 | 73.1 | 73.1 | 43.07 | <0.001 |
|  | sp*temp | 4 | 79.9 | 20.0 | 11.76 | <0.001 |
| Humic Acid Growth | sp | 7 | 3492 | 498.8 | 346.67 | <0.001 |
|  | temp | 1 | 55 | 55.1 | 38.27 | <0.001 |
|  | sp*temp | 4 | 93 | 23.2 | 16.15 | <0.001 |
| Chitinase REA | sp | 7 | 5.571 | 0.7959 | 12.813 | <0.001 |
|  | temp | 1 | 0.435 | 0.4346 | 6.997 | 0.009 |
|  | sp*temp | 5 | 5.607 | 1.1215 | 18.055 | <0.001 |
| Endoglucanase REA | sp | 7 | 49.22 | 7.031 | 37.797 | <0.001 |
|  | temp | 1 | 0 | 0.001 | 0.004 | 0.948 |
|  | sp*temp | 4 | 5.38 | 1.345 | 7.229 | <0.001 |
| β-glucosidase REA | sp | 7 | 8.77 | 1.2529 | 28.048 | <0.001 |
|  | temp | 1 | 0.715 | 0.715 | 16.005 | <0.001 |
|  | sp*temp | 4 | 0.668 | 0.1669 | 3.737 | <0.001 |
| Cellobiohydrolase REA | sp | 7 | 0.563 | 0.0805 | 5.553 | <0.001 |
|  | temp | 1 | 0.52 | 0.5198 | 35.871 | <0.001 |
|  | sp*temp | 4 | 0.348 | 0.0869 | 5.999 | <0.001 |
| Lipase REA | sp | 7 | 63.7 | 9.099 | 19.343 | <0.001 |
|  | temp | 1 | 0 | 0.001 | 0.002 | 0.961 |
|  | sp*temp | 4 | 8.88 | 2.221 | 4.721 | 0.00153 |
| Urease REA | sp | 7 | 312.85 | 44.69 | 81.814 | <0.001 |
|  | temp | 1 | 1.55 | 1.55 | 2.839 | 0.0947 |
|  | sp*temp | 5 | 43.9 | 8.78 | 16.071 | <0.001 |
| α-hemolysin REA | sp | 5 | 1.781 | 0.3563 | 3.465 | 0.0114 |
