# Supplementary material for: Comparison of the White-Nose Syndrome Agent Pseudogymnoascus destructans to Cave-Dwelling Relatives Suggests Reduced Saprotrophic Enzyme Activity
Source: PLoS One. 2014 Jan 22;9(1):e86437. doi: 10.1371/journal.pone.0086437 (PMC3899275; doi:10.1371/journal.pone.0086437)
Supplement: Table S4 — Tukey’s Highly Significant Differences Test for growth on organic acids comparing P. destructans to other species. (DOCX) [file pone.0086437.s004.docx]

**Table S4.** Tukey’s Highly Significant Differences Test for growth on organic acids comparing *P. destructans* to other species.

| Enzyme | Species | 10°C | | | | 20°C | | | |
| --- | --- | --- | --- | --- | --- | --- | --- | --- | --- |
|  |  | diff | lwr | upr | p | diff | lwr | upr | p |
| Fulvic Acid | *P. pannorum* | 8.76 | 6.29 | 11.24 | 0.00 | 10.51 | 7.95 | 13.07 | 0.00 |
|  | BL308 | 4.18 | 1.62 | 6.74 | 0.00 | 2.60 | -0.30 | 5.50 | 0.13 |
|  | BL549 | 4.62 | 2.06 | 7.18 | 0.00 | 7.00 | 4.10 | 9.90 | 0.00 |
|  | BL578 | 7.18 | 4.62 | 9.74 | 0.00 | 12.60 | 9.70 | 15.50 | 0.00 |
|  | BL606 | 3.29 | 0.73 | 5.85 | 0.00 | 5.60 | 2.70 | 8.50 | 0.00 |
|  | *P. pinophilum* | NA | NA | NA | NA | 19.19 | 16.79 | 21.58 | 0.00 |
|  | *O. maius* | NA | NA | NA | NA | 1.40 | -1.11 | 3.91 | 0.84 |
| Humic Acid | *P. pannorum* | 8.40 | 6.08 | 10.72 | 0.00 | 11.50 | 8.66 | 14.34 | 0.00 |
|  | BL308 | 3.60 | 1.28 | 5.92 | 0.00 | 4.20 | 1.53 | 6.87 | 0.00 |
|  | BL549 | 4.40 | 2.08 | 6.72 | 0.00 | 5.00 | 2.33 | 7.67 | 0.00 |
|  | BL578 | 6.50 | 4.18 | 8.82 | 0.00 | 12.40 | 9.73 | 15.07 | 0.00 |
|  | BL606 | 7.80 | 5.48 | 10.12 | 0.00 | 7.00 | 4.33 | 9.67 | 0.00 |
|  | *P. pinophilum* | NA | NA | NA | NA | 20.53 | 18.35 | 22.72 | 0.00 |
|  | *O. maius* | NA | NA | NA | NA | 2.10 | -0.22 | 4.42 | 0.12 |
