# Supplementary material for: Comparison of the White-Nose Syndrome Agent Pseudogymnoascus destructans to Cave-Dwelling Relatives Suggests Reduced Saprotrophic Enzyme Activity
Source: PLoS One. 2014 Jan 22;9(1):e86437. doi: 10.1371/journal.pone.0086437 (PMC3899275; doi:10.1371/journal.pone.0086437)
Supplement: Table S5 — Tukey’s Highly Significant Differences Test for relative enzyme activity comparing multiple fungal species to P. destructans. (DOCX) [file pone.0086437.s005.docx]

Table S5. Tukey’s Highly Significant Differences Test for relative enzyme activity comparing multiple fungal species to *P. destructans*.

| Enzyme | Species | 10°C | | | | 20°C | | | |
| --- | --- | --- | --- | --- | --- | --- | --- | --- | --- |
|  |  | diff | lwr | upr | p | diff | lwr | upr | p |
| Chitinase | *P. pannorum* | 0.17 | -0.25 | 0.59 | 0.99 | 0.51 | -0.44 | 1.45 | 0.88 |
|  | BL308 | -0.22 | -0.71 | 0.26 | 0.96 | 0.56 | 0.10 | 1.02 | 0.00 |
|  | BL549 | 0.08 | -0.38 | 0.54 | 1.00 | 0.47 | 0.02 | 0.92 | 0.03 |
|  | BL578 | 0.37 | -0.09 | 0.83 | 0.29 | 0.26 | -0.19 | 0.71 | 0.80 |
|  | BL606 | 0.11 | -0.38 | 0.59 | 1.00 | 0.42 | -0.03 | 0.87 | 0.09 |
|  | *P. pinophilum* | 0.14 | -0.35 | 0.62 | 1.00 | -0.65 | -1.13 | -0.18 | 0.00 |
|  | *O. maius* | NA | NA | NA | NA | -0.21 | -0.67 | 0.24 | 0.95 |
| Endoglucanase | *P. pannorum* | 1.96 | 1.13 | 2.79 | 0.00 | 1.84 | 1.01 | 2.67 | 0.00 |
|  | BL308 | 2.23 | 1.39 | 3.08 | 0.00 | 3.15 | 2.32 | 3.98 | 0.00 |
|  | BL549 | 2.64 | 1.79 | 3.48 | 0.00 | 2.30 | 1.47 | 3.13 | 0.00 |
|  | BL578 | 2.16 | 1.29 | 3.02 | 0.00 | 1.74 | 0.87 | 2.60 | 0.00 |
|  | BL606 | 1.73 | 0.88 | 2.57 | 0.00 | 1.60 | 0.77 | 2.43 | 0.00 |
|  | *P. pinophilum* | NA | NA | NA | NA | 1.22 | 0.39 | 2.05 | 0.00 |
|  | *O. maius* | NA | NA | NA | NA | 1.07 | 0.24 | 1.90 | 0.00 |
| β-glucosidase | *P. pannorum* | -0.79 | -1.14 | -0.44 | 0.00 | -0.79 | -1.14 | -0.43 | 0.00 |
|  | BL308 | -0.79 | -1.15 | -0.44 | 0.00 | -0.30 | -0.66 | 0.06 | 0.21 |
|  | BL549 | -0.76 | -1.11 | -0.41 | 0.00 | -0.68 | -1.03 | -0.32 | 0.00 |
|  | BL578 | -0.89 | -1.24 | -0.54 | 0.00 | -0.77 | -1.12 | -0.41 | 0.00 |
|  | BL606 | -0.84 | -1.19 | -0.49 | 0.00 | -0.69 | -1.04 | -0.33 | 0.00 |
|  | *P. pinophilum* | NA | NA | NA | NA | -0.94 | -1.29 | -0.59 | 0.00 |
|  | *O. maius* | NA | NA | NA | NA | -0.10 | -0.45 | 0.25 | 1.00 |
| Cellobiohydrolase | *P. pannorum* | -0.02 | -0.27 | 0.24 | 1 | 0.04 | -0.21 | 0.29 | 1.00 |
|  | BL308 | -0.02 | -0.27 | 0.24 | 1 | 0.14 | -0.11 | 0.39 | 0.85 |
|  | BL549 | -0.07 | -0.32 | 0.19 | 1 | 0.03 | -0.22 | 0.28 | 1.00 |
|  | BL578 | -0.03 | -0.29 | 0.22 | 1 | 0.02 | -0.23 | 0.28 | 1.00 |
|  | BL606 | -0.33 | -0.59 | -0.08 | 0.001 | 0.05 | -0.20 | 0.30 | 1.00 |
|  | *P. pinophilum* | NA | NA | NA | NA | 0.11 | -0.14 | 0.36 | 0.97 |
|  | *O. maius* | NA | NA | NA | NA | 0.03 | -0.22 | 0.28 | 1.00 |
| Lipase | *P. pannorum* | 0.18 | -0.89 | 1.26 | 1.00 | 0.37 | -0.71 | 1.45 | 1.00 |
|  | BL308 | -0.29 | -1.40 | 0.82 | 1.00 | 0.15 | -0.96 | 1.26 | 1.00 |
|  | BL549 | -0.28 | -1.60 | 1.04 | 1.00 | -0.01 | -1.08 | 1.07 | 1.00 |
|  | BL578 | 0.20 | -0.88 | 1.28 | 1.00 | -0.95 | -2.02 | 0.13 | 0.16 |
|  | BL606 | -0.54 | -1.68 | 0.60 | 0.95 | -0.03 | -1.17 | 1.11 | 1.00 |
|  | *P. pinophilum* | NA | NA | NA | NA | -1.51 | -2.58 | -0.43 | 0.00 |
|  | *O. maius* | NA | NA | NA | NA | 1.99 | 0.88 | 3.09 | 0.00 |
| Urease | *P. pannorum* | 1.41 | 0.28 | 2.55 | 0.00 | 2.18 | 0.99 | 3.37 | 0.00 |
|  | BL308 | 4.32 | 3.16 | 5.48 | 0.00 | 4.49 | 3.26 | 5.72 | 0.00 |
|  | BL549 | 4.31 | 3.08 | 5.54 | 0.00 | 2.61 | 1.42 | 3.80 | 0.00 |
|  | BL578 | 2.34 | 1.18 | 3.50 | 0.00 | 1.95 | 0.79 | 3.11 | 0.00 |
|  | BL606 | 0.71 | -0.43 | 1.84 | 0.70 | 3.06 | 1.90 | 4.22 | 0.00 |
|  | *P. pinophilum* | 0.07 | -1.27 | 1.40 | 1.00 | -0.26 | -1.42 | 0.89 | 1.00 |
|  | *O. maius* | NA | NA | NA | NA | -1.20 | -2.54 | 0.14 | 0.13 |
| Hemolysin | *P. pannorum* | -0.10 | -0.67 | 0.47 | 1.00 | NA | NA | NA | NA |
|  | BL308 | -0.02 | -0.61 | 0.57 | 1.00 | NA | NA | NA | NA |
|  | BL549 | 0.56 | -0.08 | 1.21 | 0.12 | NA | NA | NA | NA |
|  | BL578 | -0.14 | -0.76 | 0.48 | 0.98 | NA | NA | NA | NA |
|  | BL606 | 0.06 | -0.51 | 0.63 | 1.00 | NA | NA | NA | NA |
