# Supplementary material for: Comparison of the White-Nose Syndrome Agent Pseudogymnoascus destructans to Cave-Dwelling Relatives Suggests Reduced Saprotrophic Enzyme Activity
Source: PLoS One. 2014 Jan 22;9(1):e86437. doi: 10.1371/journal.pone.0086437 (PMC3899275; doi:10.1371/journal.pone.0086437)
Supplement: Table S6 — Tukeys’ Highly Significant Differences Test comparing intraspecific growth or relative enzyme activity at 20°C and 10°C. (DOCX) [file pone.0086437.s006.docx]

Table S6. Tukeys’ Highly Significant Differences Test comparing intraspecific growth or relative enzyme activity at 20°C and 10°C

| Assay | Species | diff | lwr | upr | p-value |
| --- | --- | --- | --- | --- | --- |
| Fulvic Acid Growth | BL308 | -1.578 | 4.138 | 0.982 | 0.7140641 |
|  | BL549 | 2.378 | 0.182 | 4.938 | 0.0987296 |
|  | BL578 | 5.422 | 2.862 | 7.982 | 0.000 |
|  | BL606 | 2.311 | -0.249 | 4.871 | 0.1243609 |
|  | *P. pannorum* | 1.747 | -0.315 | 3.810 | 0.1965213 |
| Humic Acid Growth | BL308 | 0.600 | -1.715 | 2.915 | 0.9999138 |
|  | BL549 | 0.600 | -1.715 | 2.915 | 0.9999138 |
|  | BL578 | 5.900 | 3.585 | 8.215 | 0.000 |
|  | BL606 | -0.800 | -3.115 | 1.515 | 0.9974901 |
|  | *P. pannorum* | 3.100 | 0.599 | 5.601 | 0.0032426 |
| Chitinase REA | BL308 | 0.783 | 0.342 | 1.224 | 0.0000011 |
|  | BL549 | 0.390 | -0.012 | 0.792 | 0.0666856 |
|  | BL578 | -0.104 | -0.506 | 0.298 | 0.999925 |
|  | BL606 | 0.316 | -0.115 | 0.748 | 0.4261036 |
|  | *P. pannorum* | 0.337 | -0.563 | 1.237 | 0.9945978 |
| Endoglucanase REA | BL308 | 0.918 | 0.222 | 1.613 | 0.0010928 |
|  | BL549 | -0.338 | -1.033 | 0.358 | 0.9411422 |
|  | BL578 | -0.419 | -1.176 | 0.338 | 0.8503903 |
|  | BL606 | -0.129 | -0.825 | 0.566 | 0.999999 |
|  | *P. pannorum* | -0.119 | -0.796 | 0.558 | 0.9999995 |
| B-glucosidase REA | BL308 | 0.491 | 0.151 | 0.831 | 0.0001805 |
|  | BL549 | 0.082 | -0.249 | 0.413 | 0.9999561 |
|  | BL578 | 0.129 | -0.203 | 0.460 | 0.9923875 |
|  | BL606 | 0.157 | -0.174 | 0.488 | 0.9525616 |
|  | *P. pannorum* | 0.008 | -0.323 | 0.339 | 1.000 |
| Cellobiohydrolase REA | BL308 | 0.156 | -0.145 | 0.256 | 1.000 |
|  | BL549 | 0.094 | -0.220 | 0.409 | 0.9995427 |
|  | BL578 | 0.055 | -0.268 | 0.377 | 0.9999997 |
|  | BL606 | 0.382 | 0.067 | 0.696 | 0.0042704 |
|  | *P. pannorum* | 0.058 | -0.267 | 0.382 | 0.9999995 |
| Lipase REA | BL308 | 0.440 | -0.695 | 1.575 | 0.9922871 |
|  | BL549 | 0.274 | -1.045 | 1.593 | 0.9999956 |
|  | BL578 | -1.144 | -2.221 | -0.067 | 0.0259211 |
|  | BL606 | 0.512 | -0.692 | 1.716 | 0.981132 |
|  | *P. pannorum* | 0.187 | -0.890 | 1.264 | 0.9999996 |
| Urease REA | BL308 | 0.171 | -1.057 | 1.400 | 1.000 |
|  | BL549 | -1.694 | -2.953 | -0.436 | 0.000722 |
|  | BL578 | -0.388 | -1.546 | 0.770 | 0.9984043 |
|  | BL606- | 2.356 | 1.224 | 3.488 | 0.000 |
|  | *P. pannorum* | 0.768 | -0.396 | 1.932 | 0.6166215 |
|  | *Penicillium pinophilum* | -0.331 | -1.668 | 1.006 | 0.9999582 |
